# Bioabsorbable interference screw versus bioabsorbable cross pins: influence of femoral graft fixation on the clinical outcome after ACL reconstruction

**DOI:** 10.1007/s00167-011-1875-4

**Published:** 2012-01-31

**Authors:** Stephan Frosch, Anne Rittstieg, Peter Balcarek, Tim Alexander Walde, Jan P. Schüttrumpf, Martin M. Wachowski, Klaus M. Stürmer, Karl-Heinz Frosch

**Affiliations:** 1 Department of Trauma Surgery, Plastic and Reconstructive Surgery, Medical University of Göttingen, Göttingen, Germany; 2 Department of Trauma and Reconstructive Surgery, Asklepios Clinic St. Georg, Hamburg, Germany

**Keywords:** Milagro screw, Interference screw, Cross pins, ACL reconstruction, Postoperative outcome

## Abstract

**Purpose:**

The aim of this study was to evaluate the clinical outcome and differences in anterior–posterior laxity of ACL reconstruction using a bioabsorbable interference screw for femoral graft fixation when compared to femoral bioabsorbable cross pin fixation.

**Methods:**

Clinical outcome was evaluated among 59 patients 1 year after arthroscopic ACL reconstruction with hamstrings graft in a prospective, non-randomised study. In 31 cases, femoral fixation of the graft was performed using a bioabsorbable interference screw. In 28 cases, two bioabsorbable cross pins were used for femoral fixation. Patients were evaluated using Tegner, Lysholm and Marshall scores, the visual analogue scale for pain and KT-1000 arthrometer measurement.

**Results:**

No significant difference (*P* ≥ 0.05) was observed at follow-up for the knee scores. The average Tegner score was 5.83 points (±2.00) for the interference screw fixation and 5.83 points (±1.24) for the cross pin fixation; the average Lysholm score was 93.58 (±5.79) to 92.72 (±6.34) points; and the average Marshall score 46.72 (±2.4) to 47.30 (±2.35) points. No significant difference was found for the visual analogue scale for pain. KT-1000 arthrometer measurement revealed a significant (*P* < 0.05) difference in the mean side-to-side anterior translation at all applied forces. At 67 N, the mean difference was 1.53 mm (±1.24) in the interference screw group and 0.47 mm (±1.18) in the cross pin group (*P* < 0.05). At 89 N, the mean differences were 1.85 mm (±1.29) versus 0.59 mm (±1.59), respectively, (*P* < 0.05), and maximum manual displacements were 2.02 mm (±1.26) versus 1.22 mm (1.18; *P* < 0.05).

**Conclusions:**

In ACL reconstruction with hamstrings graft, similar clinical results are obtained for the use of bioabsorbable cross pins when compared to bioabsorbable interference screws for femoral fixation. Cross pin fixation was superior with regard to the anteroposterior laxity as measured with KT-1000.

## Introduction

In hamstring ACL reconstruction, graft fixation is a critical factor for the healing process. Interference screws as well as cross pins are common intraosseous graft fixation techniques. Good clinical results can be achieved with both devices [1].

Bioabsorbable interference screws and metal interference screws are equally successful in graft fixation [5, 7, 20, 23], but bioabsorbable interference screws exhibit advantages due to their biodegradability [6, 8]. Disadvantages of the metal interference screws like graft irritation owing to their sharp edges, problems during potential revision procedures as well as distortion of MRI have led to the preference for bioabsorbable screws in ACL fixation [9, 14, 30].

Advantages of interference screws are as follows: (1) Reduced early motion of the graft within the tunnel, which is important for stable healing [12]; (2) Less synovial fluid in the bone tunnel, reducing possible negative effects of cytokines [13]; and (3) Avoiding the so-called bungee effect due to fixation at a point close to the tunnel entrance [13].

Disadvantages of interference screws are possible graft irritation caused by introducing the screw, a reduced bone–tendon interface and a reported slippage of the graft causing clinical failure [10, 22, 24].

A lack of replacement of bioabsorbable screws by bony tissue even after an extended period of time is reported [5, 22, 24, 27, 28], as well as inflammation of the synovium elicited by foreign body reactions [2, 25, 27].

A new development is the bioabsorbable Milagro interference screw, made of 30% β-TCP (tricalcium phosphate) and 70% PLGA (polylactic-co-glycolic acid). It was shown in a previous study [8] that due to the Milagro screw’s material properties, degradation of the screw is up to 90% after 12 months and well suited to the healing process of the ACL transplant [4, 8, 16]. In the first three to 6 months, when the graft requires stable fixation, Milagro screws displayed almost no resorption [8, 16]. Moreover, the osteoconductive properties of the material induce bony replacement of the screw, which could be advantageous in revision surgery [8].

Another common device used for ACL fixation is the biodegradable cross pin (RigidFix, DePuy Mitek, Norderstedt, Germany), which is inserted perpendicular to the tunnel and fixate the graft in the tunnel by lancing it. Several studies have shown comparable clinical results and primary stability after ACL reconstruction using either the interference screws or cross pins for securing the graft [11, 17, 29].

There is no clinical study comparing Milagro interference screw fixation with cross pin fixation on the clinical outcome and anterior–posterior laxity.

The aim of this study is to evaluate the clinical results and translational stability of ACL reconstruction with hamstring tendons using Milagro screws for femoral fixation of the graft and to compare them with the results for femoral cross pin fixation.

The hypothesis of this study is that the Milagro screw shows superior clinical outcomes as well as superior anterior–posterior laxity when compared to the cross pins in ACL graft fixation.

## Materials and methods

This prospective, non-randomised study involved fifty-nine patients who underwent hamstring ACL reconstruction. Two different femoral graft fixation methods were performed. To compare the clinical outcome of each fixation method, we separated the patients into two groups according to the fixation methods used.

Table 1 shows the demographic baseline profile of both groups.

**Table 1:** Demographic baseline profile

Variables	Cross pin group *N* = 28	Milagro group *N* = 31
Female	10	12
Male	18	19
Mean age (years)	28.2 (±8.0)	24.6 (±7.2)
Body mass index (kg/m^2^)	24.9 (±2.9)	24.6 (±3.2)
Mean interval to surgery (weeks)	11.09 (±4.0)	14.91 (±3.4)
Follow-up examination (month)	12.40 (±0.8)	12.45 (±1.1)
Accompanying injuries		
Lateral meniscus	8	7
Medial meniscus	4	3
Chondral lesion (in numbers unless otherwise labelled)	6	7

In a period of 1 year, we initially performed thirty-one ACL reconstructions using Milagro interference screws, followed by twenty-eight ACL reconstructions using the cross pin technique for femoral fixation. Tibial fixation in all patients was performed with Milagro interference screws.

Inclusion criteria are the following: (1) primary ACL rupture with the indication for an ACL reconstruction (instability signs like giving way, positive Lachman and/or pivot shift test); (2) closed epiphyseal plates.

Exclusion criteria are the following: (1) the presence of additional fractures around the knee joint; (2) previous surgery on the affected knee joint; (3) cartilage lesions ICRS grade 2 exceeding 3 cm^2^, cartilage lesions ICRS grade 3 and 4; (4) additional PCL lesions; (5) autologous chondrocyte transplantation; (6) mosaicplasty with more than one transplanted cylinder (or a cylinder >1 cm); (7) more than 50% of the medial or lateral meniscus resected; (8) patients with axis deformities or underlying diseases that resulted in physical impairment.

### Implants for graft fixation

Milagro™ interference screws (DePuy Mitek, Norderstedt, Germany) are made of 30% β-TCP (TriCalcium phosphate) and 70% PLGA (polylactic-co-glycolic acid). They are available in diameters of 7–12 mm and lengths of 23, 30 or 35 mm.

Two Cross pins (RigidFix, Ethicon, Mitek Division, Norderstedt, Germany) were used in diameter of 3.3 mm and length of 42 mm. They are made of poly-l-lactic acid (PLLA) and were used only for femoral fixation in this study.

### Surgical technique

**Fig. 1:**
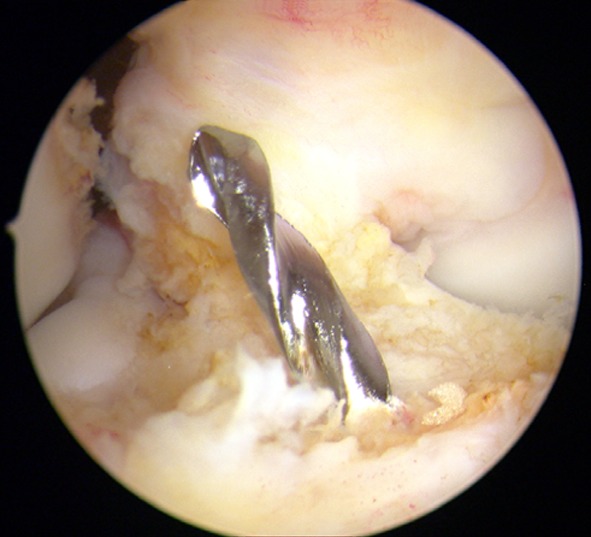
Arthroscopic picture: The tibial tunnel was placed with the tibial insertion of the ACL as reference (*right* knee)

The ACL reconstruction was performed arthroscopically by two experienced surgeons. In all cases, semitendinosus or semitendinosus combined with gracilis tendons were used as grafts, depending on the diameter and length of the tendons. The tendons were either trebled or quadrupled to reach a graft diameter of 7–8.5 mm and a graft length of 9 cm. After removal, the tendons were sutured in a whipstitch fashion and augmented on the femoral and tibial aspects. The tibial tunnel was prepared using an alignment jig, with the footprint of the ACL as reference (Fig. 1). The cortex was opened at an angle of 55° to the tibial articular surface directly above the pes anserinus.

**Fig. 2:**
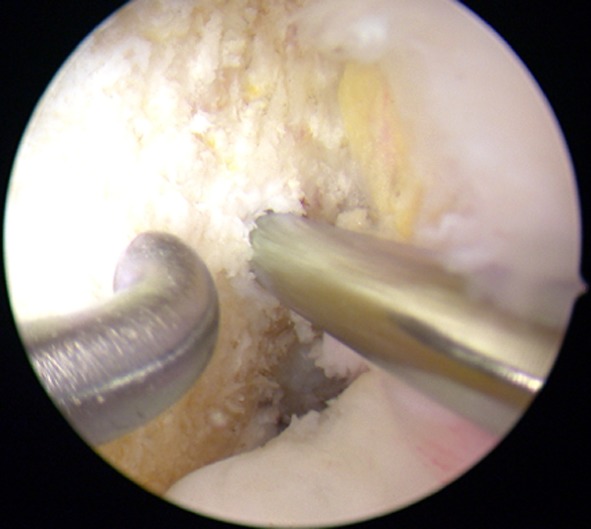
Arthroscopic picture after ACL reconstruction. The femoral tunnel was placed closer to the origin of the anteromedial bundle than to the posterolateral bundle (*left* knee)

The femoral tunnel was prepared and drilled over the anteromedial portal using an alignment jig with a 6 mm offset, related to the anatomical landmarks of the ACL, slightly more oriented to the anteromedial bundle than to the posterolateral bundle (Fig. 2). The tunnel was drilled at 120° of knee flexion.

In group one, after placing the graft in the tunnel, the Milagro screws were introduced in the femoral tunnel to fix the graft. The graft was pretensioned by moving the knee joint through its range of motion before a tibial interference screw was placed as close to the joint as possible at a knee flexion of 20°. A 23-mm femoral screw and 30-mm tibial screw were used. The bone tunnel diameter was adapted to 0.5 mm below graft height. The diameter of the Milagro screws was selected according to the bone tunnel diameter.

In group two, two cross pins were used for femoral fixation. A cross pin guide was placed in the tunnel. Two sleeves were inserted in the interlocking trocars and drilled into the lateral side of the femur towards the tunnel. After removing the guide, while the sleeves were left in place, the graft was placed in the tunnel. Then, guided by the sleeves, the cross pins were driven into the tunnel until the pins had crossed the tunnel and both ends were anchored in the bone. The subsequent steps were the same as in group one. Tibial fixation was performed using a 30-mm Milagro interference screw.

### Follow-up treatment

Care following surgery included 6 weeks of partial weight-bearing (10–20 kg) with the surgical leg using crutches. No brace should be used. The range of motion of the knee should not exceed 0–0–90° (Ext/Flex) for the first 6 weeks postoperatively.

### Clinical evaluation

The mean follow-up examination of the cross pin group was performed after 12.4 months (±0.8) and for the Milagro group after 12.45 months (±1.1). The clinical evaluation was based on three functional knee scores: the Lysholm score [26], the Tegner score [26] and the Marshall score [18]. The KT-1000 arthrometer measurement was used to evaluate the mean difference in anterior–posterior laxity (compared to the healthy contralateral knee) with a maximum measurement accuracy of 1 mm. The visual analogue scale was used to assess the level of pain during daily living (0 points = no pain, 10 points = maximal pain).

### Statistical analysis

Functional scores, anterior instability and pain score were compared between the two fixation methods using the nonparametric Mann–Whitney *U*-test. The significance level was set to alpha = 5% for all test. Analyses were performed using the software Statistica (version 9.1, StatSoft).

## Results

Thirty-nine patients were treated with an ACL reconstruction using cross pins for femoral fixation of the hamstring graft. Eleven Patients were excluded of the study corresponding to the exclusion criteria: two medial meniscus resection >50%; one lateral meniscus resection >50%; three cartilage lesions ICRS 3; two additional fractures of the proximal tibia; three previous surgeries on the affected knee joint. Twenty-eight patients were included in the study, and twenty-eight patients participated at the follow-up examination for the cross pin group.

Thirty-seven patients were treated with an ACL reconstruction using the Milagro screw for fixation of the hamstring graft. Six patients were excluded: two lateral meniscus resection >50%; three cartilage lesions of ICRS grade 3 and higher; one patient to take part in the study. All of the included thirty-one patients took part at the follow-up examination.

The follow-up percentage was 100 for the Milagro and cross pin group.

Table 1 shows the demographic baseline profile of both groups.

### Functional scores

**Fig. 3:**
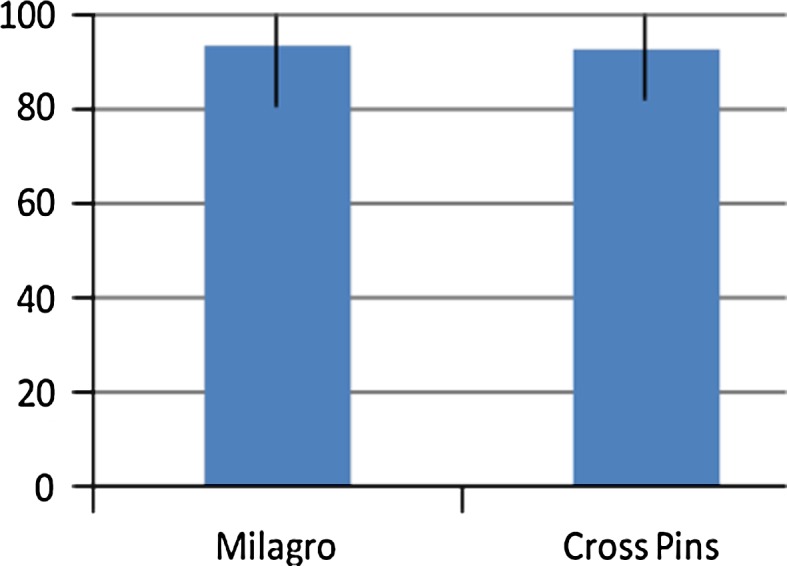
Lysholm score. No significant difference (n.s.) at the follow-up between the two groups (median values)

**Fig. 4:**
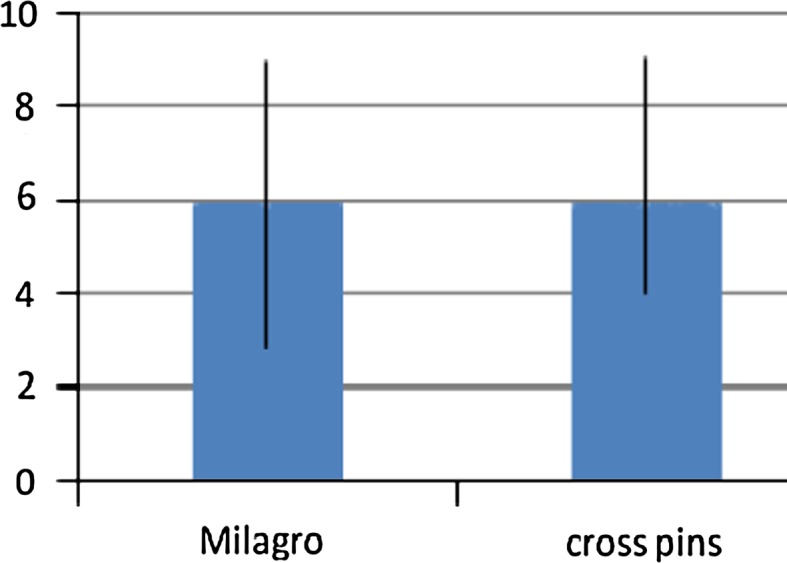
Tegner score. No significant difference (n.s.) at the follow-up between the two groups (median values)

**Fig. 5:**
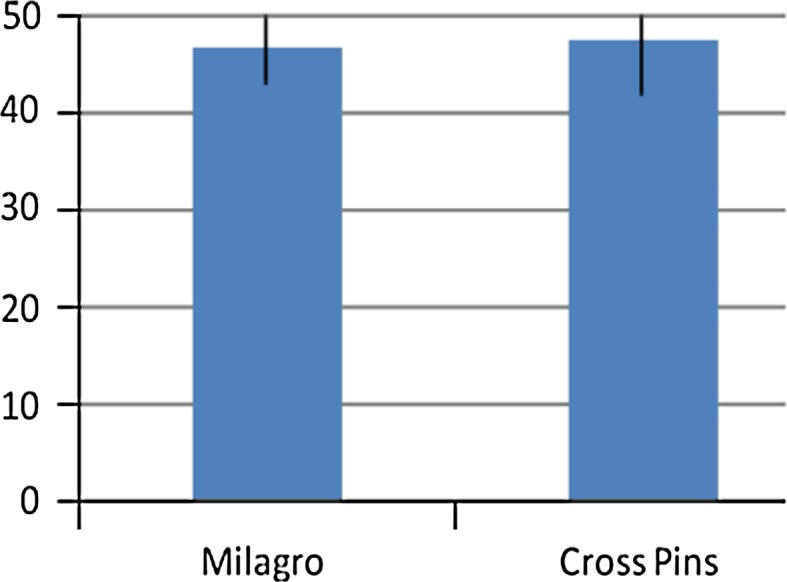
Marshall score. No significant difference (n.s.) at the follow-up between the two groups (median values)

No significant differences (n.s.) were found between the groups when comparing the femoral fixation methods with regard to the functional scores. The median values of the Lysholm score were 94 (81–100) points in the Milagro group and 94.5 (82–100) points in the cross pin group (Fig. 3). The median values of the Tegner score were 6 (3–9) points versus 6 (4–9) points (Fig. 4). The median values of the Marshall score did not differ significantly between the groups, with 47.5 (43–50) points for group one and 48 (42–50) points for group two (Fig. 5).

### KT-1000 arthrometer

**Fig. 6:**
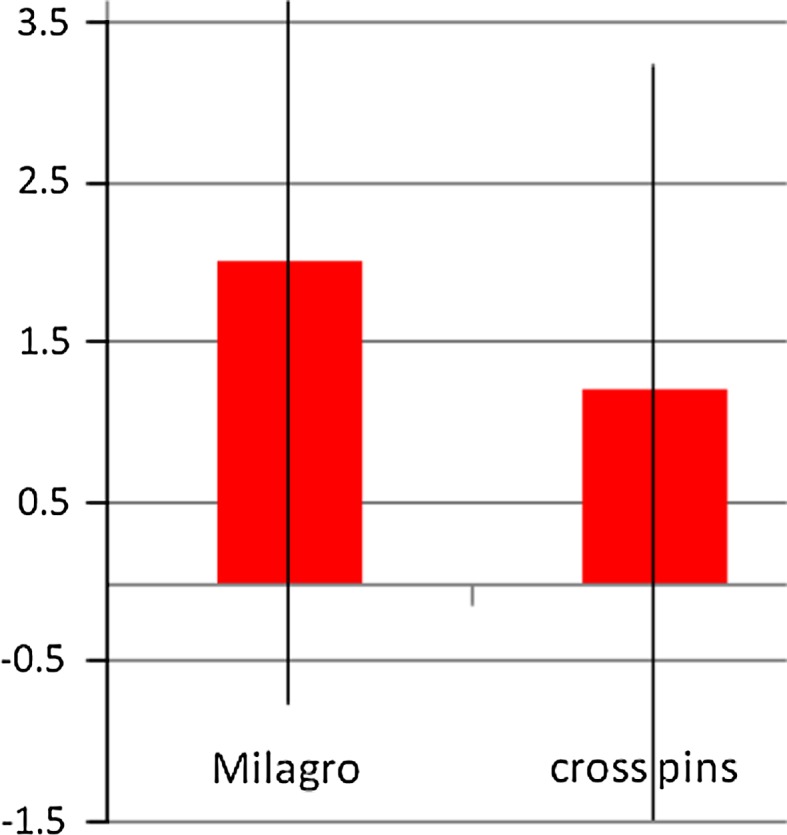
KT-1000 at max. displacement. Significant difference (*P* < 0.05) at the follow-up between the two groups

Comparing postoperative stability using the KT-1000 arthrometer revealed a significant difference between the groups (*P* < 0.05). The mean differences in anterior–posterior laxity for the Milagro group versus the cross pin group were the following: for 67 N 1.5 mm (±1.2) versus 0.5 mm (±1.2); for 89 N 1.9 mm (±1.3) versus 0.6 (±1.6); and for maximum manual displacement 2.0 mm (±1.3) versus 1.2 mm (±1.2; Fig. 6).

### Visual analogue scale for pain

Patients evaluated their pain during daily living using the visual analogue scale with a mean score of 1.1 (±1.5) for the Milagro screw group and 1.4 (±1.4) for the cross pin group. These results were not significantly different (n.s.).

### Complications

Two patients in the Milagro™ group required revision surgery due to a cyclops syndrome. No complications were found in the cross pin group.

## Discussion

The most important finding of the present study was that cross pin fixation is superior with regard to the anteroposterior laxity when compared to Milagro screw fixation as measured with KT-1000. Therefore, the hypothesis of this study that the Milagro screw shows superior clinical outcomes as well as superior anterior–posterior laxity when compared to the cross pins in ACL graft fixation must be discarded.

After ACL reconstruction, progressive rehabilitation programmes and demands of patients to participate in sports activities as early as possible require a secure and reliable fixation of the graft. Interference screws as well as cross pins show good clinical results and primary stability after ACL reconstruction [1].

During activities of daily living, forces up to 450 N stress the graft [3, 21]. Zantop et al. [29] examined the initial fixation strength of two 3.3-mm bioabsorbable pins compared to interference screws for hamstring grafts in bovine knees. In the cycle loading test, they found no significant difference under 1,000 cycles to 250 N. Remarkable finding of the study is that only grafts fixed with cross pins survived 1,000 cycles to 450 N.

Harilainen et al. [11] presented a 2-year follow-up randomised trial including 120 patients, comparing cross pin fixation and BioScrew fixation after ACL reconstruction with hamstring tendons. They found no significant difference at the 1- and 2-year follow-up evaluation at the clinical examination, knee scores (Tegner Activity Level, Lysholm, IKDC, and Patellofemoral Scores) and laxity measurements (Lachman, Pivot-shift).

According to these observations, the present study does not find any significant differences (n.s.) at the clinical outcome when comparing femoral cross pin fixation with Milagro screw fixation after ACL reconstruction. The median values of the Tegner score were 6 points (3–9) for the Milagro fixation and 6 points (4–9) for the cross pin fixation; the median values of the Lysholm score were 94 (81–100) versus 94.5 (82–100) points; and the median values of the Marshall score were 47.5 (43–50) versus 48 (42–50) points. The median values of the visual analogue scale for pain during daily living also showed no significant difference (n.s.): 1 (0–4) points versus 1.5 (0–5) points.

The KT-1000 arthrometer measurement revealed significant differences (*P* < 0.05) in the mean side-to-side anterior translation at all applied forces. At 67 N, the mean difference was 1.5 mm (±1.2) in the Milagro group 0.5 mm (±1.2) in the cross pin group. At 89 N, the mean differences were 1.9 mm (±1.3) versus 0.6 mm (±1.6), respectively, and maximum manual displacements were 2.0 mm (±1.3) versus 1.2 mm (1.2). These results support the statement of Zantop et al. [31] that interference screws provide a significant inferior biomechanical stability than cross pins do.

Although the KT-1000 arthrometer measurement revealed significant differences (*P* < 0.05) in the mean side-to-side anterior translation, there is no effect on the clinical outcome.

There are limitations of the study. First, some authors state that 1-year follow-up examination after ACL reconstruction might be too short to evaluate the postoperative outcome. In response to that, primary graft healing is completed after 12 months [8]. Direct contact is established between tendon and bone tunnel wall within 12 weeks, and bone–tendon junction takes up to 24 weeks [13]. Patients after ACL reconstruction participate in full contact sports after 7–9 months. Therefore, differences at the clinical outcome, especially differences at the anteroposterior laxity, should be revealed after 12 months. A subsequent study with a 5-year follow-up should be aimed to examine the clinical outcome.

Secondly, rotatory laxity has not been assessed. There are reports that the pivot-shift examination has significant associations with subjective symptoms and function after ACL reconstruction [15]. We did not assess the pivot-shift test, because of a limited comparability due to different muscular tension of the patients during the test and because of a high inter-observer variation [19].

Finally, we did not investigate radiographic outcomes, such as tunnel widening. There are already several studies focusing on tunnel widening after ACL reconstruction. A clinical relevance of tunnel widening could not been shown [1, 8].

The findings of this study imply an advantage of the cross pins over the interference screw in ACL fixation, and therefore we use femoral cross pins fixation as a standard procedure in ACL reconstruction.

## Conclusion

In ACL reconstruction with hamstrings graft, similar clinical results are obtained for the use of cross pins when compared to Milagro interference screws for femoral fixation. Cross pin fixation was superior with regard to the anteroposterior laxity as measured with KT-1000.
